# Do What You Ought, Come What May

**Published:** 1874-05

**Authors:** 


*" Do What You Ought, Come What May."*

Dr. ——: It is true that the Record is an independent exponent for any and every thing for the general good of the profession, still we do not feel called upon to become the special advocate of any particular cause disconnected with the scope of our journal, no matter what troubles may abide on the side of either contestants. If you feel disposed to send us a communication over your signature, setting forth the facts in the controversy alluded to, we will publish it. While we believe truth left alone will sooner or later destroy error, it is the duty of every good man to uphold it with all his might when proper occasions arise, and to obliterate error and wrong as soon as possible, the sooner that wrong is destroyed the less will be the harm done. To remove the evil is to destroy its effects. The advocates of truth have a peculiar advantage over those of error. The former rises higher continually, but the doers and endorsers of evil things sink daily more and more deeply into the mire of their own infamy. It would be better if all good men could remember that Solomon, under the inspiration of Heaven, taking into view the corruption of the human heart, said " Confidence in an unfaithful man in time of trouble is like a broken tooth and a foot out of joint." P.

